# Assessment of hepatoprotective potential of Radix Fici Hirtae on alcohol-induced liver injury in Kunming mice

**DOI:** 10.1016/j.bbrep.2018.10.003

**Published:** 2018-10-23

**Authors:** Xiaofei Feng, Kangxian Li, Fangming Tan, Mei Zhu, Jieyi Zhou, Yongjun Lai, Lingfeng Zeng, Yingting Ye, Jing Huang, Xiaosong Wu, Shasha Li

**Affiliations:** Department of Pharmacy, the First Affiliated Hospital of Jinan University, Guangzhou 510632, PR China

**Keywords:** Radix Fici Hirtae, Alcohol-induced liver injury, Hepatoprotective

## Abstract

**Objective:**

The objective of the present study was to investigate the hepatoprotective role of Radix Fici Hirtae on acute alcohol-induced liver injury in mice.

**Methods:**

The component of Radix Fici Hirtae was extracted using petroleum ether, chloroform, ethyl acetate and n-butanol and divided into three dose groups of high, medium and low according to the clinical man's normal dose of the 50 g crude drug/d (0.83 g/kg body weight). Saline in concentration of 10 mg/mL, 5 mg/mL and 2.5 mg/mL and a dose of mouse lavage (0.2 mL/10 g mouse body weight) were added to the solution. Histopathlogical analysis of liver was performed. Finally, liver protection was validated by examining the effect of aspartate aminotransferase (AST), alanine aminotransferase (ALT), alkaline phosphatase (AKP), and lactate dehydrogenase (LDH) on the hepatic function of mice in alcohol-induced liver injury model.

**Results:**

Except for group with saturated n-butyl alcohol, for the rest of the groups, pathological changes of hepatic lipid and inflammatory cells infiltration were alleviated and liver sinus was normal. As compared to model group, the concentrations of AST, ALT, AKP and LDH in chloroform groups and ethyl acetate groups were significantly decreased.

**Conclusions:**

Extracts of Radix Fici Hirtae are effective for the prevention of alcohol-induced hepatic damage in mice. The results revealed that extracts of Radix Fici Hirtae could be used as hepatoprotective agent.

## 1 Introduction

Alcohol-induced liver injury is one of the most common causes of liver diseases worldwide. Long-term alcohol consumption induces oxidative stress in the liver due to the imbalance between the prooxidant and the antioxidant systems. And then persistent oxidative stress may cause fatty liver, which can lead to inflammation, fibrosis, cirrhosis, and even liver cancer [1]. The alcohol-induced liver injury is similar to the disease of alcoholic liver disease [2]. The study of the pathogenesis of human alcoholic liver disease and screening are critical for the current study.

Radix Fici Hirtae—root of Ficus hirta Vahl, which belongs to moraceae family—is a commonly used medicine for keep healthy in south China region. It is commonly used in spleen deficiency, self-sweating, chronic bronchitis, rheumatism and is associated with anti-inflammatory and immunological functions [3], [4]. In recent years, various studies have investigated the chemical constituents, pharmacological activities and other aspects of Radix Fici Hirtae. Previous studies have shown that the main components of Radix Fici Hirtae include amino acids, sugars, steroid, vanilla bean extract, etc. [5], [6], and demonstrated its anti-oxidation, immunoregulation [7], anti-inflammatory, protective gastric mucosa activities, which improves circulation, anti-hepatotoxicity caused by cardio cerebral hypoxia ischemia and various chemicals [8], [9], [10], [11]. Moreover, Radix Fici Hirtae also has a hepatoprotective effect on N, N-dimethylformamide induced acute liver injury and cocaine-induced hepatotoxicity in mice [12], [13]. The specific hepatoprotective role of its component, however, is not clear. In order to define the pharmacodynamics material basis for hepatoprotective effect of Radix Fici Hirtae, it is imperative to screen its active sites for liver protection.

## 2 Materials and methods

### 2.1 Animals and materials

Male Kunming mice weighting 18–25 g were purchased from the Experimental Animal Centre of Guangdong Medical Laboratory, China. The animals were housed at a room temperature of 22 ± 2 ℃ with a 12 h-light and 12 h-dark cycle. Food and water were available and libitum.

Radix Fici was purchased from Baining Pharmaceutical Co., Ltd., Guangzhou，Guangdong. Assay kits for aminotransferase(ALT), aspart ateamino transferase(AST), Glutathione(GSH), alkaline phosphatase(AKP), lactate dehydrogenase(LDH) and superoxide dismutase (SOD) were purchased from Nanjing Jiancheng Biological Engineering (Nanjing, China).

### 2.2 Preparation of the different parts, the different extracts and the fractions

10 kg Radix Fici Hirtae was crushed into meal and 95% ethanol (double the amount of Radix) was added. The solution was kept in an airtight container for 6 h. The 327.3 g of alcohol extract is gain by the percolation method. Following this, the component was extracted using petroleum ether, chloroform, ethyl acetate and n-butanol. Solvent was added in the ratio of 1.5, 1.5 and 1, respectively. The amount of four extracts were 43.5 g, 5 g, 9.4 g and 49 g, respectively. The extract with 1.2 g/mL of crude drug was used, and the desired concentration of the extract was obtained by diluting with physiological saline.

### 2.3 Experimental design

After an adaptation period for seven days, 105 mice were randomly divided into 21 groups with 5mice in each group. All groups were treated once a day for consecutive 7days.

Mice received 12 mL/kg BW of 50% ethanol by gavage every 12 h for a total of 3 doses. Control mice received an isocaloric maltose solution. In the administration groups, extracts of Radix Fici Hirtae were dissolved in normal saline and gavaged simultaneously with EtOH at a different dose of 200 mg/kg, 100 mg/kg and 50 mg/kg BW, respectively. After the final ethanol dose, the mice were sacrificed and the blood and livers were collected. The serum and liver were obtained and stored immediately at −80 ℃ for further analysis.

### 2.4 Histopathological examinations

The fixed liver tissues were dehydrated in graded alcohol and embedded inparaffin. Sections of 5 mm thickness were made using a microtome and stained with haematoxylin and eosin(H&E). The histopathological changes in the liver were observed under a light microscope (Nikon Eclipse TE2000-U, Japan).

### 2.5 Estimation of serum ALT, AST, AKP and LDH activity

Serum ALT, AST, AKP and LDH activity were measured colorimetrically using a diagnostic kit according to the instructions provided.

### 2.6 Statistical analysis

Data were analyzed with SPSS 19.0 software. Results were shown as mean±SD. All data were analyzed by one-way analysis of variance, and the differences between groups were established by Dunnett test. A value of p < 0.05 was considered statistically significant.

## 3 Results

### 3.1 HE staining in liver tissue of mice

We observed the pathological changes of liver tissues by HE staining. As can been seen from the normal group, liver cells were arranged neatly without degeneration, steatosis or necrosis.

In the control group, liver tissues radiated around the central veins, and liver cells were arranged neatly without degeneration, steatosis or necrosis. In the model group, spotty necrosis was observed mainly around the central vein of the hepatic lobule. We also observed medium to severe steatosis in the liver lobule and lymphocyte infiltration in portal area, showing obvious injuries in the liver tissue.

The liver lobules of mice in model group (M-G) were not distinguishable by optical microstructure, as the lobules were destroyed during liver cell transformation from fat. Over 95% of liver cells were fatty; hyperplasia of numerous fibrous septa was observed in the liver of some animals. It is indicative of liver injury in mice. The border of liver cell of mice in the control group (N-G) is clear, with distinct shape of cell; whereas the liver cells of hepatic lobule of mice in the model group (M-G) were arranged radially.

In the chloroform group and residual aqueous solution group, the morphology and boundary of cells are intact and clear. However, a blurred cell boundary and inflammatory infiltration were observed in the other groups.

**Fig. 1:**
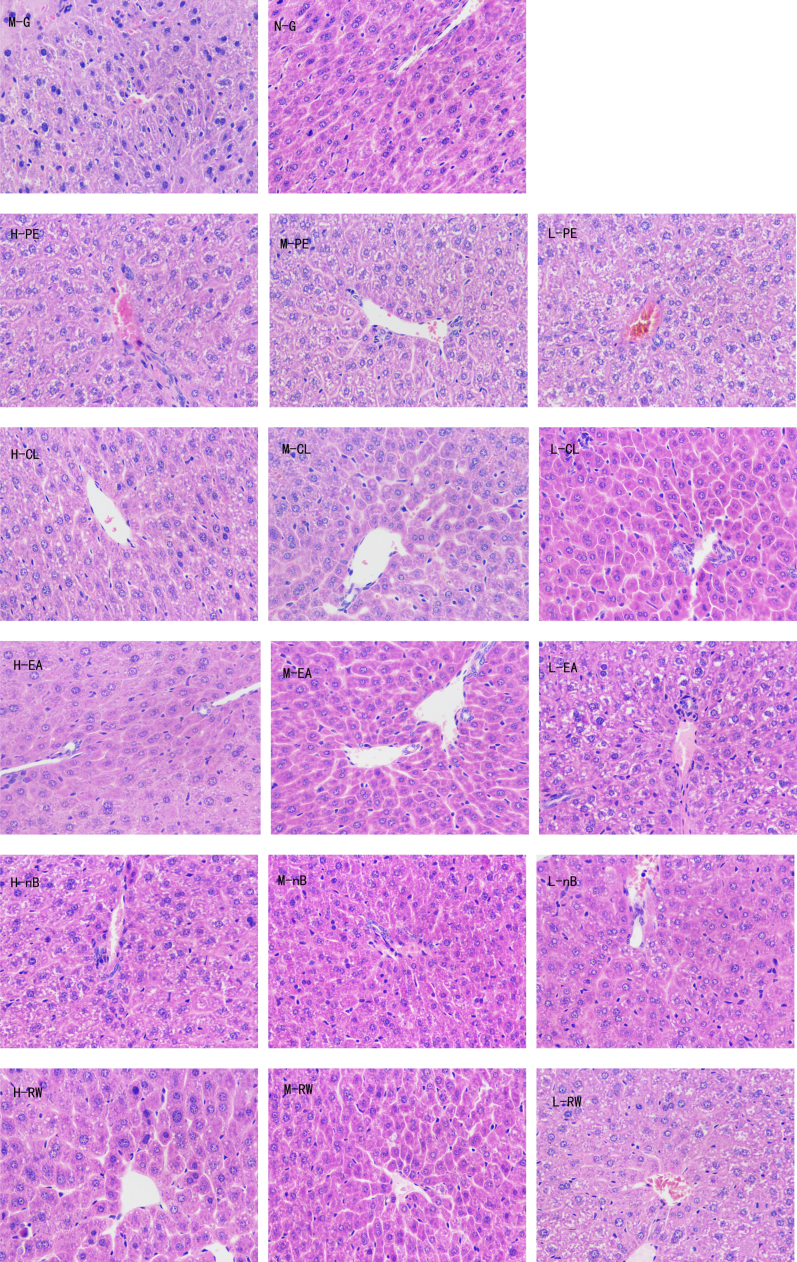
Changes in hepatic pathology (HE×400). Note: M-G：model group, N-G：normal control group, H-PE：high dose group of petroleum ether, M-PE：middle dose group of petroleum ether, L-PE：low dose group of petroleum ether, H-CL：high dose group of chloroform, M-CL：middle dose group of chloroform, L-CL：low dose group of chloroform, H-EA：high dose group of ethyl acetate, M-EA：middle dose group of ethyl acetate, L-EA：low dose group of ethyl acetate, H-nB：high dose group of saturated n-butyl alcohol, M-nB：middle dose group of saturated n-butyl alcohol, L-nB：low dose group of saturated n-butyl alcohol, H-RW：high dose group of residual aqueous solution, M-RW：middle dose group of residual aqueous solution, L-RW：low dose group of residual aqueous solution.

Except for group with saturated n-butyl alcohol, for the rest of the groups, pathological changes of hepatic lipid and inflammatory cells infiltration were alleviated and liver sinus was normal. These findings indicated that the regeneration of liver cells presents different levels of liver protection (Fig. 1).

### 3.2 Estimation of serum AST, ALT, AKP and LDH activity

**Fig. 2:**
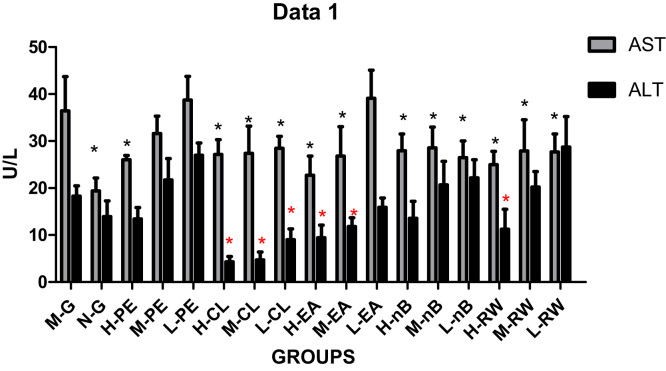
Change in AST and ALT levels in different groups.

The results of serum AST are shown in Table 1 and Fig. 2. A significant increased AST levels was observed in the model group, as compared to the control mice (p < 0.05). A significant decreased AST levels was observed in all the chloroform groups, saturated n-butanol groups and residual aqueous solution groups, as compared to the model group(p < 0.05). Furthermore, in the ethyl acetate group with high and medium dose, a significant decreased AST levels was observed (p < 0.05), while there was no significant difference in the low dose group. Besides, there was a significant decreased AST levels in the petroleum ether group with high dose (p < 0.05).

**Table 1:** Change of indexes of the serum in each dose group (n = 6).

Group	AST(U/L)	ALT(U/L)	AKP(king U/100 mL)	LDH(U/L)
M-G	36.45 ± 7.29	18.27 ± 2.23	34.39 ± 2.20	1930.72 ± 473.45
N-G	19.41 ± 2.73*	13.94 ± 3.33	27.42 ± 3.59*	1016.97 ± 279.19*
H-PE	26.04 ± 0.91*	13.43 ± 2.43	18.52 ± 2.56*	1328.11 ± 349.25*
M-PE	31.59 ± 3.74	21.74 ± 4.52	29.01 ± 1.95	1040.95 ± 239.50*
L-PE	38.74 ± 5.05	26.99 ± 2.64	32.19 ± 6.75	1448.07 ± 111.04*
H-CL	27.14 ± 3.17*	4.3 ± 1.14*	21.92 ± 2.50*	531.65 ± 111.86*
M -CL	27.38 ± 5.79*	4.74 ± 1.64*	23.24 ± 4.17*	782.85 ± 124.86*
L-CL	28.47 ± 2.56*	9.03 ± 2.29*	22.3 ± 4.88*	1953.38 ± 175.64
H-EA	22.73 ± 4.06*	9.42 ± 2.69*	23.81 ± 4.67*	868.47 ± 249.40*
M-EA	26.82 ± 6.25*	11.82 ± 1.85*	21.75 ± 5.55*	1311.63 ± 222.15*
L-EA	39.11 ± 6.00	15.88 ± 2.03	32.94 ± 7.18	1616.64 ± 325.62
H-n-B	27.95 ± 3.57*	13.55 ± 3.65	23.41 ± 2.25*	434.49 ± 44.57*
M-n-B	28.57 ± 4.41*	20.68 ± 4.99	27.31 ± 5.40*	915.99 ± 196.68*
L-n-B	26.46 ± 5.54*	22.17 ± 3.84	23.28 ± 2.83*	1462.64 ± 276.39*
H-RW	24.99 ± 2.83*	11.23 ± 4.25*	24.66 ± 3.90*	646.98 ± 167.60*
M-RW	27.91 ± 6.62*	20.24 ± 3.30	23.54 ± 4.14*	599.21 ± 97.18*
L-RW	27.70 ± 3.81*	28.82 ± 6.51	20.95 ± 5.00*	857.51 ± 210.07*

In comparison with the model group, the groups with decreased ALT were observed in all the chloroform groups, ethyl acetate groups with high and medium dose and high dose group of residual aqueous solution (p < 0.05). However, there was no significant difference in other groups (Table 1, Fig. 2).

**Fig. 3:**
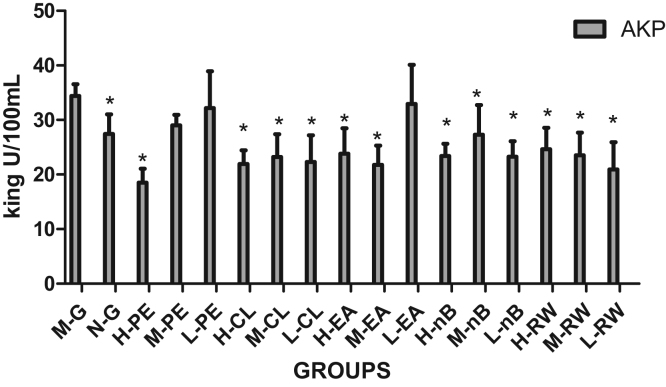
Change in AKP levels in different groups.

The results of serum AKP are shown in Table 1 and Fig. 3. A significant decreased AKP levels was observed in all the chloroform groups, saturated n-butanol groups and residual aqueous solution groups, as compared to the model group (p < 0.05). In the ethyl acetate group with high and medium dose, a significant decreased AST levels was observed (p < 0.05), while there was no significant difference in the low dose group. Besides, there was a significant decreased AST levels in the petroleum ether group with high dose (p < 0.05).

**Fig. 4:**
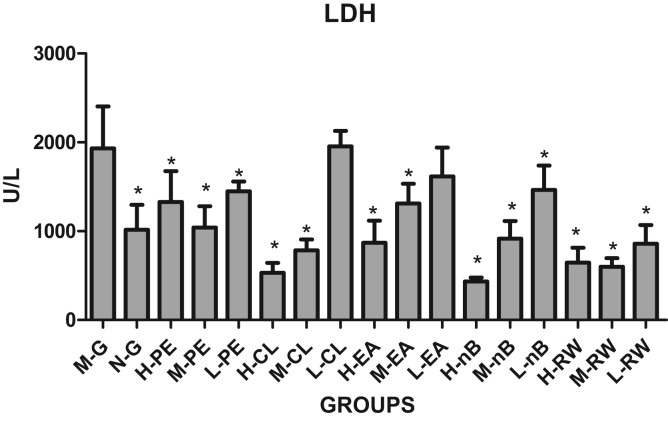
Change in LDH levels in different groups.

The results of serum LDH are shown in Table 1 and Fig. 4. Compared with the model group, there was a significant decreased LDH levels in all the petroleum ether groups, saturated n-butanol groups and residual aqueous solution groups (p < 0.05). And in the chloroform group and ethyl acetate group with high-medium dose, there was also a significant decrease, as copared to the model group (p < 0.05), while there was no significant decrease in the low dose group.

## 4 Discussion

Our results showed that in model group, the concentration of AST, ALT, AKP and LDH in serum of mice with moderate liver damage was increased significantly. In addition, inflammatory infiltrates and fat cavitation were observed in the liver tissue.

Except for group with saturated n-butyl alcohol, for the rest of the groups, the histopathological lesion of the liver tissue is markedly reduced as compared to model group, thereby indicating that Radix Fici Hirtae has significant protective functions on mice with acute liver injury caused by alcohol in mice.

Aspartate aminotransferase (AST) is used as medical clinical index for liver function examination. AST primarily exists in liver cell mitochondria. The concentration of AST in serum is increased during necrosis or liver damage [14]. Alanine aminotransferase (ALT), another indicator of liver damage, primarily exists in liver, heart and skeletal muscle. The level of ALT is increased in blood during tissue damage and necrosis. Thus, increased levels of these enzymesare a marker in detecting acute and chronic liver injury [15]. Therefore, it is possible that Radix Fici Hirtae extracts through reduction of necrosis that results from its antioxidant property, prevents the leakage of these enzymes into the blood and decreases these serum levels.

Alkaline phosphatase (AKP) belongs to a phosphoglycerase family. Lactate dehydrogenase (LDH), belongs to glycolyase family, exists in the cytoplasm of all tissues, but mainly in kidney. In the present study, alcohol-induced liver toxicity has demonstrated considerable increase in serum ALP and LDH. Due to extracts of Radix Fici Hirtae administration, the parameters were decreased significantly [16].

ALT, AST and AKP are mainly found in the cytoplasm. The intracellular transaminases enter the blood and increase the serum activity during liver damage [17], [18], [19]. Therefore, the transaminases are considered as efficient biomarkers of liver tissue damage. Our study showed that Radix Fici Hirtae can reduce the concentration of ALT, AST, AKP and LDH in serum, which is caused by acute alcohol-induced hepatic injury. In comparison with the model group, it has been found that the concentrations of ALT, AST, AKP and LDH in serum are significantly reduced in groups with different dose of chloroform groups and ethyl acetate groups.

## 5 Conclusion

In conclusion, the results of this study demonstrate that extracts of Radix Fici Hirtae are effective for the prevention of alcohol-induced hepatic damage in mice. Moreover, the possible mechanisms of hepatoprotective effect maybe attributed due to the freer adical scavenging effect and inhibition of lipid peroxidation. The results revealed that extracts of Radix Fici Hirtae could be used as hepatoprotective agent. However, the active components or the active parts was not clear. To identify the effective parts and clarify the precise mechanism responsible for the hepatoprotective effects of Radix Fici Hirtae, further studies are being conducted by our research group.
