# Supplementary material for: Assessment of hepatoprotective potential of Radix Fici Hirtae on alcohol-induced liver injury in Kunming mice
Source: Biochem Biophys Rep. 2018 Oct 23;16:69–73. doi: 10.1016/j.bbrep.2018.10.003 (PMC6202661; doi:10.1016/j.bbrep.2018.10.003)
Supplement: Supplementary file 1 — Supplementary material [file mmc1.doc]

Conflict of Interest

The authors declare no competing financial interest.
